# Impact of Cyanidin-3-Glucoside on Gut Microbiota and Relationship with Metabolism and Inflammation in High Fat-High Sucrose Diet-Induced Insulin Resistant Mice

**DOI:** 10.3390/microorganisms8081238

**Published:** 2020-08-14

**Authors:** Fei Huang, Ruozhi Zhao, Min Xia, Garry X. Shen

**Affiliations:** 1 Departments of Food and Human Nutritional Sciences, Internal Medicine, University of Manitoba, Winnipeg, MB R3T2N2, Canada; huangf1@myumanitoba.ca; 2 Diabetes Research Group, Department of Internal Medicine, University of Manitoba, 835-715 McDermot Ave, Winnipeg, MB R3E 3P4, Canada; Ruozhi.Zhao@umanitoba.ca; 3 School of Public Health, Sun Yat-Sen University, Guangzhou 510275, China; xiamin@mail.sysu.edu.cn

**Keywords:** cyanidin-3-glucoside, Saskatoon berry, high fat-high sucrose diet-induced insulin-resistant mice, gut microbiota, inflammation

## Abstract

The present study assessed the effects of freeze-dried cyanidin-3-glucoside (C3G), an anthocyanin enriched in dark-red berries, compared to Saskatoon berry powder (SBp) on metabolism, inflammatory markers and gut microbiota in high fat-high sucrose (HFHS) diet-induced insulin-resistant mice. Male C57 BL/6J mice received control, HFHS, HFHS + SBp (8.0 g/kg/day) or HFHS + C3G (7.2 mg/kg/day, equivalent C3G in SBp) diet for 11 weeks. The HFHS diet significantly increased plasma levels of glucose, cholesterol, triglycerides, insulin resistance and inflammatory markers. The HFHS + SBp diet increased the *Bacteroidetes/Firmicutes* (B/F) ratio and relative abundance of *Muriculaceae* family bacteria in mouse feces detected using 16S rRNA gene sequencing. The HFHS + SBp or HFHS + C3G diet attenuated glucose, lipids, insulin resistance and inflammatory markers, and increased the B/F ratio and *Muriculaceae* relative abundance compared to the HFHS diet alone. The relative abundances of *Muriculaceae* negatively correlated with body weight, glucose, lipids, insulin resistance and inflammatory mediators. Functional predication analysis suggested that the HFHS diet upregulated gut bacteria genes involved in inflammation, and downregulated bacteria involved in metabolism. C3G and SBp partially neutralized HFHS diet-induced alterations of gut bacteria. The results suggest that C3G is a potential prebiotic, mitigating HFHS diet-induced disorders in metabolism, inflammation and gut dysbiosis, and that C3G contributes to the metabolic beneficial effects of SBp.

## 1. Introduction

Diabetes has become one of the most common metabolic disorders worldwide, and the trend of this surge is continuing. Nine out of ten diabetic patients in adults have type 2 diabetes (T2D), which is characterized as insulin resistance and is often associated with obesity [1]. Genetic factors and multiple environmental factors are implicated in the etiology of T2D. However, the precise mechanism or treatment for T2D remains unclear [2]. A Western pattern diet enriched with fat or sugar plays a crucial role in the epidemic of T2D in the world [3]. T2D is associated with low-grade chronic inflammation [4]. Accumulating lines of evidence suggest that gut microbiota plays an important role in the development of diabetes, obesity and inflammation [5]. Foods are the most common modulator of gut microbiota. Western pattern diet-induced gut dysbiosis is associated with obesity and T2D [6].

Saskatoon berry (*Amelanchier alnifolia Nutt.*), a type of fruit-bearing shrub, belongs to the *Rosaceae* family in *Amelanchier* genus and is also known as Juneberry and service berry [7]. It natively grows in the southern Yukon, the Prairies in Canada and the west northern regions of the United States of America, and has more recently been planted in Europe [8,9]. Saskatoon berry fruits contain more polyphenols, including anthocyanins, compared to other common berries, such as strawberry or blueberry [10]. At least four types of anthocyanins have been detected in Saskatoon berries, among which cyanidin-3-galactoside and cyanidin-3-glucoside (C3G) are most abundant, accounting for about 61–74% and 18–21%, respectively [8,11].

Saskatoon berry fruits possess abundant antioxidant capacity [12,13]. Previous studies by our group demonstrated that Saskatoon berry powder (SBp) was capable of reducing vascular inflammation in leptin receptor-knockout (db/db) diabetic mice. Supplementation of C3G resulted in 3-times higher anti-inflammation activity than C3Ga in cultured endothelial cells [11,14]. Our recent study demonstrated that supplementation of 5% SBp (~8.0 g/kg/day) attenuated high fat-high sucrose (HFHS) diet-induced hyperglycemia, hyperlipidemia, insulin resistance, inflammation and gut dysbiosis in mice [15]. However, the effect of the administration of C3G on the composition of gut microbiota of mice fed with a HFHS diet, and the relationship between the changes in gut microbiota, metabolism and inflammatory mediators in mice receiving a HFHS diet supplemented with C3G have not been documented.

The present study compared the effects of supplementation of C3G and SBp on circulating glucose, lipids, insulin, inflammatory markers and gut microbiota in a HFHS diet-sensitive rodent model, C57 BL/6J mice. Differences in the impact of C3G and SBp supplementation on gut microbial profile and the potential function of the microbial genes in mice fed with a HFHS diet were further investigated using 16S ribosome ribonucleic acid (rRNA) gene sequencing and bioinformatics approaches.

## 2. Materials and Methods

### 2.1. Animals

Male C57 BL/6J mice (*n* = 32, 6 weeks of age) were obtained from the Jackson Laboratory (Bar Harbor, ME, USA). Mice were housed in standard plastic cages in an air-conditioned room with an alternative 12 h day/night light cycle and received regular mouse chow and tap water for 1 week to stabilize. The protocols of animal experiments have been approved by the Animal Protocol and Ethics Committee in the University of Manitoba.

### 2.2. Dietary Intervention

Mice were randomized into 4 groups (*n* = 8/group, four in a cage) and received one of following diets for 11 weeks: (1) control group receiving a D12450K control diet from Research Diets (New Brunswick, NJ, USA), containing 4.3% of diet, 19.2% of protein and 67.3% of carbohydrates in dry mass without an addition of sucrose, (2) the HFHS group fed with a HFHS diet (D12492, Research Diets) containing 35% of fat, 26% of protein and 26% of carbohydrates, including 9% sucrose, (3) the SBp group receiving a HFHS diet supplemented with 8.0 g/kg body weight of SBp daily and (4) the C3G group fed with a HFHS diet supplemented with 7.2 mg/kg/day of C3G (equal to the amount of C3G containing 8.0 g/kg SBp). SBp was prepared from lyophilized Smoky Saskatoon berries obtained from the Prairie Lane Saskatoon (Portage, MB, Canada) and stored at −80 °C [11]. Purified C3G was obtained from Polyphenols (Sandnes, Norway).

### 2.3. Animal Monitoring and Sample Collection

Body weights and food intake of animals was assessed every 2 weeks until 10 weeks after the start of the dietary experiment. Blood (200 μL) was collected from mouse saphenous veins every 2 weeks after an overnight fasting for measuring levels of plasma glucose to monitor the development of diabetes. Mice were euthanized at 11 weeks after the onset of the dietary experiment via the inhalation of 5% isoflurane to reduce the pain of the animals. Blood was withdrawn subsequently through heart puncture.

### 2.4. Analyses of Metabolic Variables

The levels of plasma glucose and cholesterol of mice were analyzed using Sekisui Diagnostics SL reagent kits (Charlottetown, PE, Canada). Plasma levels of triglycerides were measured using BioAssay Systems reagents (Hayward, CA, USA). Insulin levels in plasma were assessed using enzyme-linked immunosorbent assay (ELISA) kits from EMD Millipore (Billerica, MA, USA) for insulin. The homeostatic model assessment of insulin resistance (HOMA-IR) of the mice was calculated from plasma insulin and glucose from simultaneously withdrawn blood samples as previously described [16].

### 2.5. Measurement of Circulating Inflammatory Markers

Monocyte chemotactic protein-1 (MCP-1) and plasminogen activator inhibitor-1 (PAI-1) were measured using ELISA kits from Thermo Fisher Scientific (Ottawa, ON, Canada) for MCP-1 and Oxford Biomedical Research (Oxford, MI, USA) for PAI-1, respectively.

### 2.6. Fecal Sample Collection

Mice were housed in a singly housed cage with fresh bedding overnight at the 10th week after the start of the dietary experiment. Fecal pellets were collected from individual cages and stored in separated tubes at −80 °C before further analysis.

### 2.7. Extraction and Sequencing of Bacteria DNA

DNA extraction was achieved using a PowerFecal DNA Isolation Kit (QIAGEN, Germantown, MD). DNA was quantified using a NanoDrop 2000 spectrophotometer (Thermo Scientific, Boston, MA, USA). Bacteria DNA in mouse feces was amplified using polymerase chain reaction (PCR) with modified primers containing 515F (5′-GTGYCAGCMGCCGCGGTAA) and 926R (5′-CCGYCAATTYMTTTRAGTTT) targeting the V4–V5 region of bacterial DNA. A high-throughput Hamilton Nimbus Select robot and Coastal Genomics analytical gels were run to verify the quality of PCR products. Failed amplicons with spurious bands were repeatedly amplified after modifying conditions for PCR until qualified bands were produced. The PCR amplicons were normalized by using a high-throughput Charm Biotech Just-a-Plate 96-well normalization kit, then pooled to construct a library and quantified before sequencing on an Illumina MiSeq platform in the Integrated Microbiome Resource in the Dalhousie University [17].

### 2.8. Bioinformatic Analysis and Statistics

Freshly generated raw data of gut microbiota in the form of a fastq file was demultiplexed according to the barcode sequences, followed by trimming using Cutadapt (version 1.17) to remove primers. Trimmed reads were imported as artifacts into an open-source bioinformatics pipeline of decentralized microbiome analysis package Quantitative Insights Into Microbial Ecology 2 (QIIME2, version: 2018. 8) [18]. After joining of paired-end reads and filtering out of low-quality reads, amplicon sequence variants (ASVs) were obtained through DADA2 workflow. Taxonomies were assigned to ASVs using a Naive-Bayes approach and SILVA database. Diversity metrics (Core-metrics-phylogenetic) within QIIME2 were used for evaluating α- and β-diversity. Differences between data from multiple groups were examined using the analysis of variance (ANOVA) and post-hoc Tukey test. Significant difference was set at *p* < 0.05. Relative abundances of ASVs assigned with taxonomy in the feature table were correlated with physical and clinical parameters. The Phylogenetic Investigation of Communities by Reconstruction of Unobserved States (PICRUSt) was used to identify differences in predictive metagenome function. Functions of microbial genes were predicted with the use of Galaxy web application, and Kyoto Encyclopedia of Genes and Genomes [19].

## 3. Results

### 3.1. Effects of HFHS Diet and Supplementation of C3G on Body Weights and Food Intake of Mice

**Figure 1:**
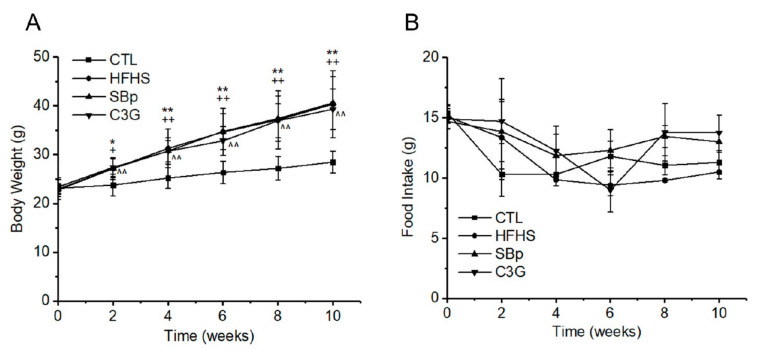
Effects of Cyanidin-3-glucoside (C3G) supplemented in a high fat-high sucrose (HFHS) diet on the body weight and food intake of mice. Male C57 BL/J6 mice (6 weeks of age) were randomized into 4 groups and received the following diets for 11 weeks: control (CTL) group: control diet, HFHS group: HFHS diet, Saskatoon berry powder (SBp) group: SBp (8.0 g/kg/day) supplemented in the HFHS diet, C3G group: C3G (7.2 mg/kg/day) supplemented in the HFHS diet. Body weights and food intake were measured every two weeks up to 10 weeks. (**A**) Body weights, (**B**) daily food intake. The values are expressed as mean ± standard deviation (SD) g (*n* = 8/group). *, **: *p* < 0.05 or 0.01 HFHS versus CTL group, +, ++: *p* < 0.05 or 0.01 SBp versus CTL group, ^^: *p* < 0.01 C3G versus CTL group.

Significant increases in body weights between mice fed with the control diet and the HFHS diet with and without a supplementation of SBp or C3G were detected at ≥2 weeks after the start of the intervention (*p* < 0.05 or 0.01). The trend of body weight increase in mice receiving the HFHS diet continued throughout the experiment. No significant difference was detected among mice fed with the HFHS diet with or without a supplementation with SBp or C3G (Figure 1A). Daily food intake of all mice was measured at onset and every 2 weeks during the dietary experiment until the 10th week after the start of the dietary intervention. No significant difference in food intake was detected among various groups receiving different experimental diets in the present study (Figure 1B).

### 3.2. Effects of C3G on Glucose and Lipid Metabolism in HFHS Diet-Induced Obese Mice

**Figure 2:**
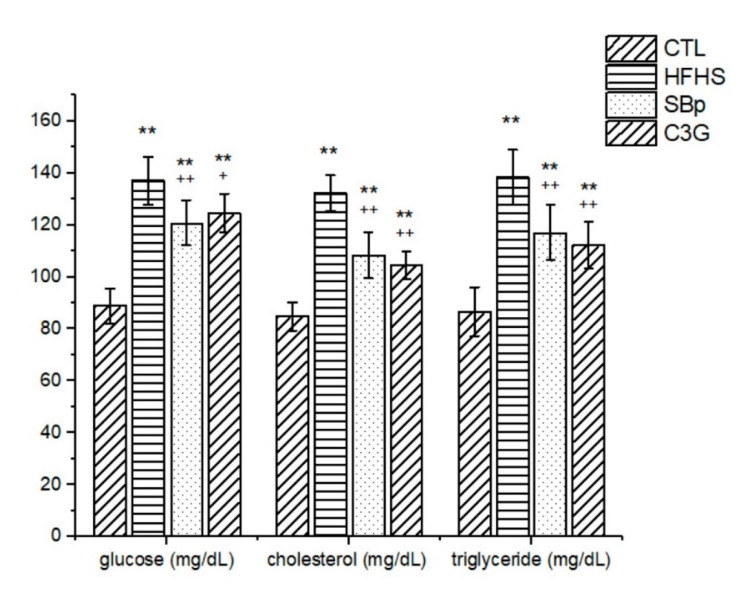
Levels of glucose, cholesterol and triglycerides in plasma of mice fed with HFHS diets supplemented with or without C3G. The dietary regimen was the same as described in the legend of Figure 1. Fasting plasma glucose, cholesterol and triglycerides were measured biochemically using assay kits. Values are expressed as mean ± SD mg/dL (*n* = 8/group). **: *p* < 0.01 versus the control (CTL) group; +, ++: *p* < 0.05 or 0.01 versus the HFHS group.

The HFHS diet significantly increased the levels of fasting plasma glucose (FPG) in mice compared to the control diet (*p* < 0.01). The supplementation with SBp or C3G in the HFHS diet significantly lowered FPG, compared to that in HFHS alone (*p* < 0.05 or 0.01). However, the levels of FPG in the SBp or C3G group were still significantly higher than that in the control group. No significant difference in FPG was found between the SBp and C3G groups. The HFHS diet also significantly elevated the levels of cholesterol and triglycerides in plasma compared to the control diet (*p* < 0.01). Plasma cholesterol or triglyceride levels in both the SBp and C3G groups were significantly lower than that in the HFHS group (*p* < 0.01). Cholesterol and triglyceride levels in the plasma of mice receiving the HFHS diet supplemented with SBp or C3G were significantly higher than those in the control group (*p* < 0.01). No significant difference in plasma cholesterol or triglycerides was detected between the SBp and C3G groups (Figure 2).

### 3.3. Effects of C3G on Fasting Plasma Insulin and Insulin Resistance in Mice Receiving the HFHS Diet

**Figure 3:**
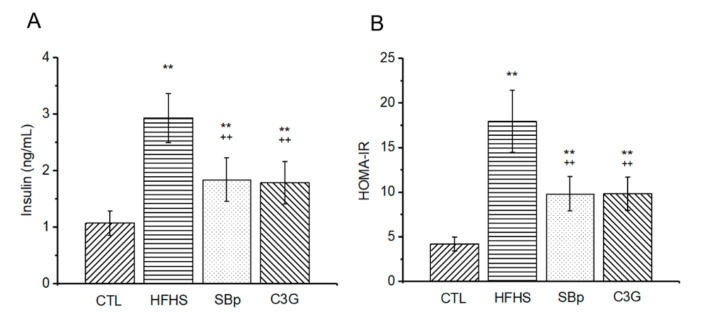
Effects of HFHS diets supplemented with C3G on insulin and insulin resistance in mice. The experimental regimen was described in the legend of Figure 1. The levels of fasting plasma insulin (ng/mL) were measured at indicated time points (**A**). Homeostatic model assessment of insulin resistance (HOMA-IR) was calculated according to the levels of glucose and insulin in the same plasma samples (**B**). Values are expressed as mean ± SD (*n* = 8/group). **: *p* < 0.01 versus the control (CTL) group; ++: *p* < 0.05 or 0.01 versus the HFHS group.

Insulin resistance was assessed using HOMA-IR generated from the levels of FPG and insulin in the plasma samples simultaneously withdrawn from mice. The levels of plasma insulin and HOMA-IR in the HFHS group were significantly higher than that in the control group (*p* < 0.01). Supplementation with SBp and C3G significantly reduced the levels of insulin and HOMA-IR compared to that in mice fed with the HFHS diet alone (*p* < 0.01). The levels of insulin and HOMA-IR in the SBp or C3G group were still higher than that in the control group (*p* < 0.01). No significant difference in insulin or HOMA-IR was detected between the SBp and C3G groups (Figure 3A,B).

### 3.4. Effects of HFHS Diet and the Supplementation of C3G on Circulating Inflammatory Markers in Mice

**Figure 4:**
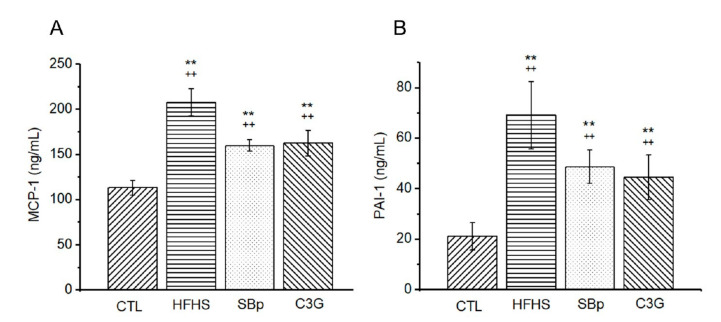
Levels of inflammatory regulators in plasma of mice receiving the HFHS diet supplemented with C3G. The experimental regimen was described in the legend of Figure 1. The levels of monocyte chemotactic protein-1 (MCP-1) and plasminogen activator inhibitor-1 (PAI-1) were analyzed in plasma collected before tissue harvesting using enzyme-linked immunosorbent assay (ELISA) kits for mouse MCP-1 (**A**) or PAI-1 (**B**). Values are expressed as mean ± SD ng/mL (*n* = 8/group). **: *p* < 0.01 versus the control (CTL) group; ++: *p* < 0.05 or 0.01 versus the HFHS group.

The HFHS diet elevated the levels of MCP-1 and PAI-1, two inflammatory markers, in the plasma of mice (*p* < 0.01). The supplementation of SBp or C3G in the HFHS diet significantly reduced the circulating levels of MCP-1 and PAI-1 compared to the HFHS diet in mice (*p* < 0.01). The levels of the inflammatory markers in mice receiving the SBp or C3G diets were still significantly higher than that in the control group (*p* < 0.01). No significant difference in plasma MCP-1 or PAI-1 was detected between mice receiving the HFHS diet supplemented with SBp or C3G (Figure 4A,B).

### 3.5. Impact of C3G Supplementation on Gut Microbiota in HFHS Diet-Fed Mice

**Figure 5:**
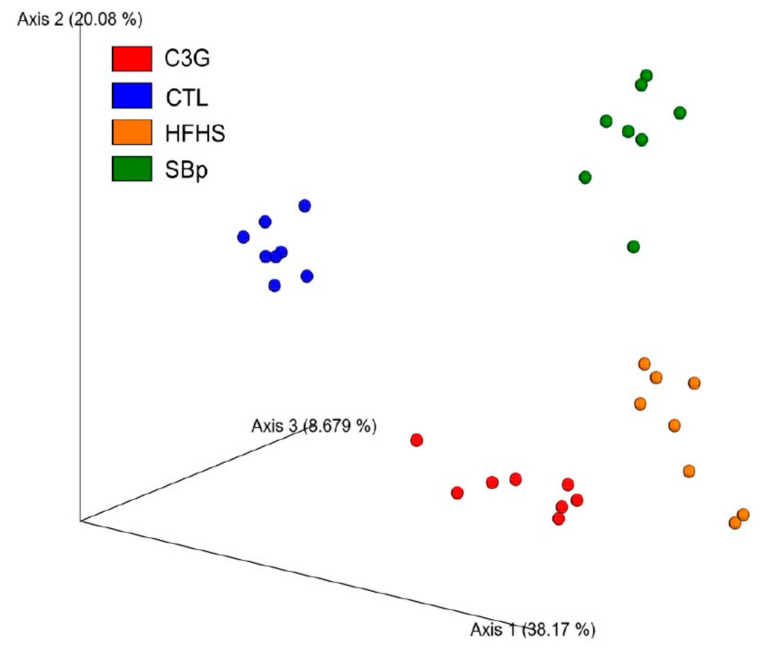
Effect of the HFHS diet supplemented with C3G on β-diversity of gut microbiota in mice. The experimental regimen was described in the legend of Figure 1. Principle component analysis (PCA) was based on Bray–Curtis dissimilarities between all sample sets (weighted by taxon abundance).

The results of β-diversity analysis demonstrated that the gut microbial compositions in the stool of mice from the four dietary groups were well separated in principal component analysis (Figure 5). No significant difference in α-diversity variables was detected in mice receiving different diets (Shannon index, chao1). Table 1 demonstrates that *Bacteroidetes* and *Firmicutes* represented the vast majority phylum bacteria in mouse feces. Mice fed with the HFHS diet had lower abundances of *Bacteroidetes*, *Actinobacteria*, *Proteobacteria* and *Verrucomicrobia phylum bacteria,* but a higher relative abundance of *Firmicutes* phylum bacteria compared to mice receiving the control diet (*p* < 0.05 or 0.01). Supplementation with SBp or C3G to the HFHS diet augmented the relative abundance of *Bacteroidetes* and reduced that of *Firmicutes* in mice feces (*p* < 0.01). The relative abundance of *Actinobacteria i*n the stool of the C3G group was the only type of phylum bacteria significantly different from that in SBp group (*p* < 0.05).

**Figure 6:**
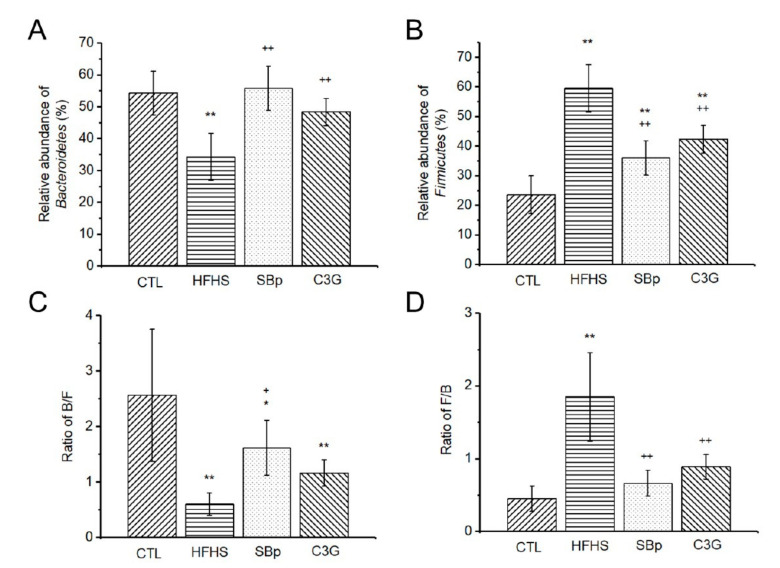
Effects of HFHS diet supplemented with or without the relative abundance of *Bacteroidetes* and *Firmicutes* and their ratios. The experimental regimen was described in the legend of Figure 1. (**A**) Relative abundance (%) of *Bacteroidetes* in gut microbial composition. (**B**) Relative abundance (%) of *Firmicutes* in gut microbial composition. (**C**) Ratio of *Bacteroidetes* over *Firmicutes* (B/F) in gut microbiota. (**D**) Ratio of *Firmicutes* over *Bacteroidetes* (F/B) in gut microbiota. Values are expressed as mean ± SD (%) (*n* = 8/group). *, **: *p* < 0.05 or 0.01 versus the control (CTL) group; +, ++: *p* < 0.05 or 0.01 versus the HFHS group.

The relative abundance of *Bacteroidetes* phylum bacteria in mice with HFHS diets was significantly lower than that in mice fed with the other three types of diets (*p* < 0.01, Figure 6A). The relative abundance of *Firmicutes* phylum bacteria in the HFHS group was significantly higher compared to that in the other three groups (*p* < 0.01, Figure 6B). The HFHS group also had a significantly lower ratio of *Bacteroidetes*/*Firmicutes* (B/F) and a higher *Firmicutes*/*Bacteroidetes* ratio compared to that in the control, SBp or C3G groups (*p* < 0.05 or 0.01). The SBp group had a significantly higher B/F ratio and lower F/B ratio compared to the HFHS group. C3G supplementation induced a significantly lower F/B ratio (*p* < 0.01), but not B/F ratio, compared to the HFHS group (Figure 6C,D).

**Figure 7:**
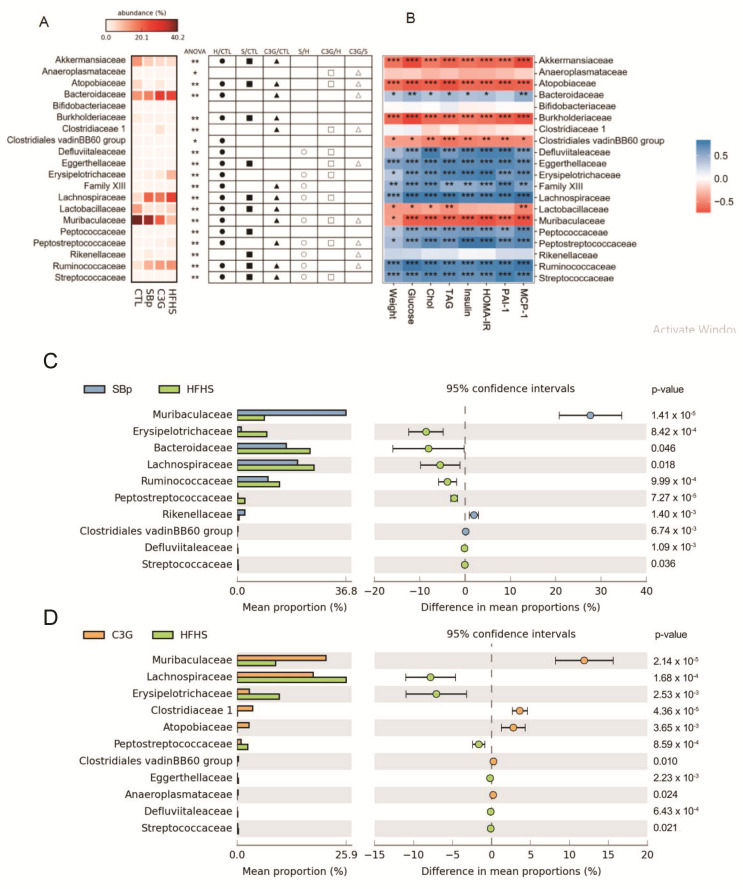
Effect of four HFHS (H) diets supplemented with C3G on the relative abundance of gut family bacteria. The experimental regimen was described in the legend of Figure 1. (**A**) Statistical differences among mice with different diets (analysis of variance (ANOVA) and post-hoc Tukey test), (**B**) correlation heatmap of relative abundance of gut microbiota on family level with physiological and biochemical parameters, (**C**) extended error bar plot (STAMP tool) showing difference in mean relative abundance between the SBp (S) group and HFHS group, (**D**) mean proportion and difference in mean proportion of family bacteria (STAMP tool) between the C3G group and the HFHS group (mean ± SD). ∗: *p* < 0.05 in overall ANOVA result, ●: *p* < 0.05 in the HFHS (H) group versus the control (CTL) group (H/CTL), ■: *p* < 0.05 in the SBp (S) group versus the CTL group (S/CTL), ▲: *p* < 0.05 in the C3G group versus the CTL group (C3G/CTL), ○: *p* < 0.05 in the S group versus the H group (S/H), □: *p* < 0.05 in the C3G group versus the H group (C3G/H), △: *p* < 0.05 in the C3G group versus the S group (C3G/S). *, **, ***: *p* < 0.05 or 0.01 or 0.001 in positive (blue) or negative (red) correlations between the abundance of each gut family bacteria and physiological or biochemical variables.

Statistical differences in the relative abundances of all types of gut family bacteria, except that of *Bifidobacteriaceae,* were detected among mice receiving various diets (*p* < 0.01, Figure 7A). Mice fed with the HFHS diet had evidently higher relative abundances in *Erysipelotrichaceae* or *Lachnospiraceae* and lower relative abundance in *Muribaculaceae* family bacteria compared to that in the other groups (heatmap in Figure 7A). Supplementation of SBp or C3G decreased the relative abundance of *Lachinospiraceae*, and increased that of *Muribacculaceae* compared to HFHS diet (*p* < 0.05). However, the relative abundances of several family bacteria in the SBp group were different from that in the C3G group (see open triangles in Figure 7A). The heatmap (Figure 7B, right) demonstrated correlations between the relative abundance of family bacteria and some diabetes-related biochemical parameters. The abundances of *Defluviitaleaceae*, *Eggerthellaceae*, *Erysipelotrichaceae*, *Family XIII*, *Lachnospiraceae*, *Peptpcoccaceae*, *Peptostreptococcaeae*, *Rununococcaceae* and *Streptococcaceae* family bacteria were positively correlated with body weights, glucose/lipid metabolism and inflammatory markers in the mice, while *Clostridiaceae 1*, *Clostridiales vadinBB60 group*, *Muribaculaceae* and *Lactobacillaceae* were negatively correlated with the physical, metabolic and inflammatory variables. The relative abundance of *Muribaculaceae* in mice in the HFHS group was the lowest among all groups. Both SBp and C3G supplementations significantly increased the relative abundances of *Muribaculaceae* family bacteria compared to the HFHS group.

The greatest difference in the mean proportion of family bacteria between the HFHS group and the SBp or C3G groups was *Muribaculaceae* family bacteria (Figure 7C,D). The mean proportion of *Muribaculaceae* in the HFHS group was significantly lower than that in the SBp or C3G groups (*p* < 0.001). The mean proportion of *Lachnospiraceae* was significantly higher in the HFHS group and lower in the SBp and C3G groups (*p* < 0.05 or 0.001). A similar pattern of changes was detected in the mean proportion of *Erysipelotrichaceae*, which was higher in the HFHS group but lower in the SBp and C3G group. The mean proportion of *Ruminococcaceae* was significantly higher in the HFHS group compared to the SBp group (*p* < 0.001, Figure 7C), while no significant difference in the mean proportion of *Ruminococcaceae* was detected between the HFHS and C3G groups (Figure 7D).

### 3.6. Functional Predication of the Changes in Gut Microbial Genes Induced by the HFHS Diet with and without C3G Supplementation

**Figure 8:**
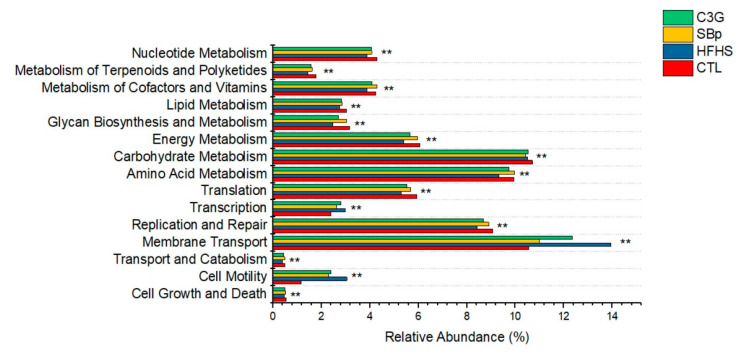
Effects of HFHS diets supplemented with and without C3G on metagenome functional activity in gut microbiota based on the Phylogenetic Investigation of Communities by Reconstruction of Unobserved States (PICRUSt). The experimental regimen was the same as described in legend of Figure 1. Differences in relative abundance (%) in each selected pathway among various dietary groups are in the form of a bar plot. Values are expressed as mean value (*n* = 8/group). **: *p* < 0.01, showing ANOVA results among the four groups.

Function prediction using PICRUSt analysis demonstrated the impact of the HFHS diet and that supplemented with SBp or C3G on the relative abundances of genes of gut microbes in 15 classes of predicted functions in mice (*p* < 0.01, ANOVA, Figure 8). Microbial genes related to membrane transport were the most abundant in the stool of mice in the present study. At least two types of ASV (operational taxonomic unit or OTUs) distribution among the cellular functions in mice receiving different dietary intervention were found: (1) the HFHS diet resulted in the highest microbial abundances compared to that in the control group, and the supplementation with SBp or C3G attenuated the effect of the HFHS diet, and (2) mice in the control group had more ASVs than in the HFHS group, and the supplementation of SBp or C3G substantially increased ASVs compared to the HFHS diet, but still relatively less than that in the control group. Examples for the first type of cellular functions include membrane transport and cell motility, which may be implicated in inflammation. The second type of cellular functions included the metabolism for carbohydrates, amino acids, lipids, cofactors, vitamins, energy generation, replication and repair, which may potentially contribute to metabolism. It is noticed that transcription was activated, but the translation pathway was suppressed in mice receiving the HFHS diet. SBp or C3G supplementation attenuated the effect of the HFHS diet in transcription or translation in mice. The findings suggest that SBp and C3G may neutralize the effect of the HFHS diet in inflammation and promote metabolism in mice via the modulation of gut microbiota.

## 4. Discussion

The major novel findings generated from the present study include: (1) the supplementation of C3G significantly reduced HFHS diet-induced hyperglycemia, hypercholesterolemia, hypertriglyceridemia, insulin resistance, inflammatory markers and gut dysbiosis in mice in similar extents as those treated with SBp containing a comparable amount of C3G, (2) the HFHS diet supplemented with C3G increased the abundance of *Bacteroidetes* phylum bacteria and decreased the abundance of *Firmicutes* phylum bacteria compared to the HFHS diet alone, (3) C3G supplementation increased the abundance of gut *Muribaculaceae* family bacteria in comparable intensity as SBp supplementation, but the influence of C3G on several other types of family bacteria differed from that in mice receiving SBp. The abundance of *Muribaculaceae* bacteria in mouse feces was negatively correlated with body weights, FPG, lipids and inflammatory markers in the mice. (4) The results of functional prediction analysis suggest that the supplementation of C3G reduced the abundance of gut microbial genes involved in inflammation and enhanced gut microbial genes involved in the metabolic processes in mice receiving the HFHS diet.

Previous studies demonstrated that C3G increased the translocation of glucose transporter-4 in skeletal muscle through the activation of insulin and the AMP-activated protein kinase pathway in mice [20]. C3G also augmented glucose-induced insulin secretion in INS-1E pancreatic β-cells and glucose uptake in HepG2 hepatocytes [21]. C3G inhibited high-glucose-induced cholesterol accumulation in HK-2 kidney epithelial cells through the activation of the LXRα pathway [22]. C3G-rich Bayberry extract reduced mitochondrial reactive oxygen species production and necrosis of INS-1 cells, and lowered blood glucose in streptozotocin-induced diabetic mice [23]. Previous studies in our laboratory demonstrated that C3G inhibited glycated low-density lipoproteins-induced NADPH oxidase activation, mitochondrial dysfunction and cell viability in cultured vascular endothelial cells [24]. The inhibitory effect of C3G on endoplasmic reticulum stress markers in endothelial cells was at least 3-times stronger than the most abundant anthocyanin, cyandin-3-galacotoside, in SBp [14]. SBp supplementation significantly reduced HFHS diet-induced hyperglycemia, hypercholesterolemia, hypertriglyceridemia and insulin resistance in C57 BL/6J mice [15]. The results of the present study demonstrated that C3G induced similar hypoglycemic and hypolipidemic effects to SBp containing a comparable amount of C3G in HFHS diet-induced insulin-resistant mice. The findings of the present study are consistent with the previous studies on the effects of Saskatoon berry and C3G in glucose and lipid metabolism reported by our and other groups [11,20,23]. The findings suggest that C3G potentially plays a critical role in the metabolic benefits of SBp in glucose and lipid metabolism.

A recent study demonstrated that C3G administration normalized gut dysbiosis induced by a food contaminant, 3-chloro-1,2-propanediol, in rats, which was characterized by increased abundances of *Lachnospiraceae* and *Actinobaceria* phylum bacteria compared to 3-chloro-1,2-propanediol alone [25]. C3G is relatively stable in stomach and intestine [26], and it is converted to active metabolites, protocatechuic, vanillic and *p*-coumaric acids in gastrointestinal rats with the assistance from gut microbiota [27]. The present study demonstrated that C3G inhibited HFHS diet-induced gut dysbiosis in mice. Both C3G and SBp significantly increased the abundance of *Bacteroidetes* phylum bacteria and *Muribaculaceae* family bacteria. *Muribaculaceae* is a newly re-named family of bacteria under phylum *Bacteroidetes,* previously known as *S24-7* or *Homeothermaceae* family. Over 600 species of *Muribaculaceae* family bacteria were inferred. Mouse was the most common host of *Muribaculaceae,* but it was also found in human, pig and non-human primates [28]. The functions of *Muribaculaceae* family bacteria have been mainly studied in mice to date. *Muribaculaceae* negatively influenced cellular processes involved in the development of chronic inflammatory bowel disease in mice [29]. An anti-diabetic agent, Acarbose, elongated lifespan in mice, which was associated with an increased abundance of *Muribaculaceae* and propionate, a short-chain fatty acid (SCFA), in feces [30]. Reduced levels of SCFA and relevant bacteria in the gut were associated with T2D patients and reversed by a high-fiber diet [31]. The findings of the present study suggest that *Muribaculaceae* family bacteria may be the targets of C3G and SBp in gut microbiota, which may contribute to the beneficial effects of C3G or SBp on metabolism and inflammation in mice.

Although the impact of supplementation of C3G and SBp on the relative abundances of gut *Bacteroidetes* phylum bacteria and *Muriculaceae* family bacteria in HFHS diet-fed mice was comparable, the effects of C3G on the compositions of 8 families of gut bacteria were significantly different from that associated with SBp (Figure 7A). For example, the relative abundance of *Clostriaceae 1* was significantly increased in the C3G group compared to that in the HFHS group, but its relative abundance was not significantly different between the SBp and HFHS groups (Figure 8). The results suggest that both SBp and C3G supplementation upregulated the relative abundance of *Muriculaceae* family bacteria, a type of gut bacteria negatively correlated with diabetes and inflammation, in mice gut, but their effects on the regulation of other family bacteria were not consistent, which indicates that the difference in other family bacteria may result from other components in SBp.

Functional prediction analysis helps to predict the potential of changes in gut microbiome based on metabolic pathways. Previous studies demonstrated that a high-fat diet reduced lipid metabolism, including short-chain fatty acids and the substrates for hepatic gluconeogenesis [32]. A recent study found that strawberry supplementation induced marked changes in functional potentials of microbial composition in db/db mice [33]. The present study has demonstrated that the HFHS diet upregulated the genes of gut microbes with predicted functions related to inflammation and downregulated microbial genes related to lipids, carbohydrates, amino acid, cofactors and vitamins. SBp or C3G supplementation to the HFHS diet tends to normalize the impact of the HFHS diet on metabolism and inflammation in gut microbiota in mice. We noticed that the HFHS diet increased the abundance of bacterial genes related to transcription but decreased the genes of bacteria contributing to protein translation in mice, which possibly results from the inhibitory effect of the HFHS diet on the translation of genes related to the metabolism, such as enzymes and cofactors. SBp and C3G supplementation neutralized the contradictory effects of the HFHS diet on microbial genes related to transcription and translation in mice (Figure 8). The outcome of the functional prediction supports the metabolic and inflammatory changes induced by the HFHS diet and the resilient capacity of SBp or C3G in metabolism in HFHS diet-fed mice in the present study.

## 5. Conclusions

The present study demonstrated that C3G supplementation resulted in similar beneficial effects compared to SBp containing an equal amount of C3G on HFHS diet-induced disorders in metabolism, inflammation and gut microbiota in mice. The results of functional predication analysis indicated that C3G reversed the changes in microbe genes related to metabolism and inflammation induced by the HFHS diet in mice. The findings suggest that C3G and SBp are potential prebiotics, which may mitigate Western diet-induced disorders in metabolism, inflammation and gut dysbiosis. The safety and proper dosage of C3G in humans remain to be determined. Saskatoon berry is a recognized fruit and can be used as a functional fruit or food supplement. The results of the present study may help to design clinical studies to investigate the efficacy of C3G and functional foods containing high amounts of C3G, such as Saskatoon berry, for the prevention and management of T2D in humans.

## Figures and Tables

**Table 1:** Effect of different diets on the abundances of gut microbiota on phylum level.

Phylum Bacteria	CTL (%)	HFHS (%)	SBp (%)	C3G (%)
Actinobacteria	5.66 ± 2.75	0.26 ± 0.11 **	0.24 ± 0.15**	2.85 ± 1.83 *,+,^
Bacteroidetes	54.28 ± 6.91	34.25 ± 7.36 **	55.80 ± 6.95 ++	48.36 ± 4.31 ++
Firmicutes	23.59 ± 6.41	59.53 ± 8.00 **	36.01 ± 5.73 **,++	42.34 ± 4.63 **,++
Proteobacteria	2.36 ± 0.71	0.68 ± 0.20 **	0.81 ± 0.21 **	0.63 ± 0.26 **
Tenericutes	0.21 ± 0.23	0.20 ± 0.14	0.24 ± 0.16	0.46 ± 0.34
Verrucomicrobia	13.88 ± 3.81	4.96 ± 3.23 **	6.78 ± 2.27 **	5.29 ± 1.81 **
Others	0.02 ± 0.02	0.12 ± 0.09 *	0.11 ± 0.09	0.08 ± 0.03

Male C57 BL/J6 mice (6 weeks of age) were randomized into 4 groups and received following diets for 11 weeks: control (CTL) group: low-fat diet; HFHS group: HFHS diet; SBp group: SBp (8.0 g/kg/day) supplemented in the HFHS diet; C3G group: C3G (7.2 mg/kg/day) supplemented in the HFHS diet. Values in the tables were expressed in mean ± SD (% of total gut microbiota, *n* = 8/group). *, **: *p* < 0.05 or 0.01 versus control group; +, ++: *p* < 0.05 or 0.01 versus HFHS group; ^: *p* < 0.05 versus SBp group.

## Declarations

Ethics approval and consent: The animal experimental protocols have been approved by the Animal Protocol and Ethics Committee in the University of Manitoba (AC-11328, approved on 27 September 2017). R.Z. and F.H., a graduate student, received required training and his involvement in the animal experiments has been approved. Consent for human data is not applicable to the present study.

## Author's Information

F.H. is a graduate student in the Department of Food and Human Nutritional Sciences in the University of Manitoba, supervised by G.X.S.

## Abbreviations

ANOVA	analysis of variance
ASVs	amplicon sequence variants
B/F	Bacteroidetes/Firmicutes
C3G	cyanidin-3-glucoside
db/db	leptin receptor knockout (mice)
ELISA	enzyme-linked immunosorbent assay
FPG	fasting plasma glucose
HFHS	high fat-high sucrose
HOMA-IR	homeostatic model assessment of insulin resistance
MCP-1	monocyte chemotactic protein-1
PAI-1	plasminogen activator inhibitor-1
PCR	polymerase chain reaction
PICRUSt	Phylogenetic Investigation of Communities by Reconstruction of Unobserved States
QIIME2	Quantitative Insights Into Microbial Ecology 2
rRNA	ribosomal ribonucleic acid
SBp	Saskatoon berry powder
T2D	type 2 diabetes
